# By the Way

**Published:** 1910-07-23

**Authors:** 


### BY THE WAY.

### THE SALE OF SECRET REMEDIES.

THERE is some satisfaction in knowing that the sale of secret remedies in this country is not increasing; in fact, the popularity of this class of medicament appears to be declining very slightly, notwithstanding the tremendous efforts of manufacturers to induce the public to dose itself with compounds of unknown composition. The revenue from medicine stamp duty during the year ended March 31, 1910, was about £2,500 less than in the preceding year, and nearly £22,000 less than in the year before that. Nevertheless, the sale of quack nostrums is still enormous, enriching the revenue in the last financial year by nearly £313,000. Since the duty on each article of the value of 1s. or under is 1½d., it follows that, supposing every package sold were of such value, the total sale of dutiable medicines amounted to over fifty million packages. A fair proportion of the patent medicines sold are of a higher price and subject to a higher duty, so that it is not possible to ascertain the precise number of packages actually sold, but a very good idea of the extent of the traffic can be obtained from the statement that the revenue derived from this source is equivalent to 50,000,000 shilling bottles or boxes of secret remedies. During the past ten years the revenue from the sale of patent medicines has amounted to nearly three and a quarter millions sterling, representing over 500,000,000 articles of the selling price of 1s. each. In other words, the British public has spent during the last ten years between twenty and twenty-five million pounds on stuff the bulk of which is either useless or noxious rubbish.

### THE TRIALS OF A COUNTRY DOCTOR.

UNDER the heading "Souvenirs d'un médecin de campagne," Dr. G. Duchesne relates in the *Concours Médical* an unpleasant experience which fell to his lot in 1865. The tongue of scandal was not idle in the village regarding the relations of his female servants with a certain Père Martin, a childless widower, owner of a magnificent property situated in a fertile valley between well-wooded hills. This individual called on the author one morning and asked him to go and attend to a farm hand of his who had given birth the evening before to an illegitimate child. Mother and child were found to be doing well, but two days later the same person came to announce the death of the infant and asked for a death certificate. The author's suspicions were aroused, and on arriving at the farm he found the mother in bed with the dead child, which had obviously died of suffocation. The only history that could be elicited was that the child had cried much early in the night, but that subsequently he had quieted down, after which he began to grow cold. The mother had made no attempt to get help for fear of waking the household. The certificate was consequently refused, and a subsequent post-mortem examination confirmed the author's diagnosis. The mother was sentenced to three months' imprisonment for "homicide par imprudence." On his way home from the court the author had to seek police protection from a valet-lover of the woman, who threatened to throw him into the river. Then commenced an organised campaign of slander and menaces on the part of the owner of the property and his domestics, as the result of which the author began to doubt whether he had done the right thing in exposing a matter which could have been hushed up easily without anyone being the wiser. It was not long, however, before he found out by roundabout ways that, had he kept silence, public opinion would have taken the matter up, and that a quasi-anonymous account of the whole proceedings would have been put before the authorities, in which event his position would have been decidedly uncomfortable, as he would have run the risk of being charged with culpable ignorance, if not with guilty complicity. He therefore draws a moral from his anecdote, which is that the facts of any case must be carefully examined, and if there be any irregularity or breach of the law, this must, after due regard to professional secrecy, be reported to the proper authority. In such cases the straightforward policy is the only one to follow, for it prevents possible accusations of ignorant or purposive complicity, from which it is always difficult to clear oneself.

### THE SNUFF-BOX.

ACCORDING to the *Journal de Médecine de Bordeaux*, the taking of snuff with fingers which are often by no means clean, and which may be the playground of germs of all kinds, is a detestable habit, contrary alike to the rules of hygiene and to what is commonly called cleanliness. What a number of diseases, such as folliculitis, otitis media, pharyngitis, enlarged tonsils, not to mention pulmonary and digestive disorders, could be avoided if this agreeable but dirty habit were given up! The journal is led to this outburst of indignation by the case of a carman who acquired an unenviable reputation for witchcraft (and this, be it noted, in an age when the existence of witches and sorcerers is no longer believed in) as the result of his prodigality in distributing the contents of his snuff-box to all-comers. While he himself and his most intimate friends experienced no ill-effects from the tobacco dust, the upper lip and anterior nasal fossæ of those less inured to the mixture were quickly attacked by crops of pimples distributed over the hair follicles. No doubt this immunity of those accustomed to the inhalations was effected by the development of an increased resistance to microbial infection, or mayhap the mixture of nasal mucus with the tobacco powder gave rise to a sort of anti-body to the staphylococci and other micro-organisms of folliculitis. Experiments *in vitro* can alone determine the interesting question of the probable attenuation of these organisms by tobacco. On the whole, therefore, it is perhaps for the best that the habit of "snuffing" is gradually dying out in this country.

